# Moscatilin Induces Apoptosis in Human Head and Neck Squamous Cell Carcinoma Cells via JNK Signaling Pathway

**DOI:** 10.3390/molecules25040901

**Published:** 2020-02-18

**Authors:** Eunji Lee, Ah-Reum Han, Bomi Nam, Ye-Ram Kim, Chang Hyun Jin, Jin-Baek Kim, Young-Gyu Eun, Chan-Hun Jung

**Affiliations:** 1 Department of Otolaryngology-Head and Neck Surgery, School of Medicine, Kyung Hee University, Seoul 02447, Korea; eun_ji0819@naver.com (E.L.); ygeun@khu.ac.kr (Y.-G.E.); 2 Advanced Radiation Technology Institute, Korea Atomic Energy Research Institute, Jeongeup-si, Jeollabuk-do 56212, Korea; arhan@kaeri.re.kr (A.-R.H.); bomi1201@kaeri.re.kr (B.N.); yrkim327@kaeri.re.kr (Y.-R.K.); chjin@kaeri.re.kr (C.H.J.); jbkim74@kaeri.re.kr (J.-B.K.); 3 Jeonju AgroBio-Materials Institute, Jeonju-si, Jeollabuk-do 54810, Korea

**Keywords:** moscatilin, *Dendrobium*, head and neck squamous cell carcinoma, FaDu, apoptosis

## Abstract

Dendrobii Herba is an herbal medicine that uses the stems of *Dendrobium* species (Orchidacea). It has been traditionally used to treat fever, hydrodipsomania, stomach disorders, and amyotrophia. In our previous study, a bibenzyl compound, moscatilin, which is isolated from Dendrobii Herba, showed potent cytotoxicity against a FaDu human pharyngeal squamous carcinoma cell line. Prompted by this finding, we performed additional studies in FaDu cells to investigate the mechanism of action. Moscatilin induced FaDu cell death by using 5 μM of concentration and by mediating apoptosis, whereas cell proliferation following treatment with 1 μM of moscatilin was not suppressed to the same levels as by the anti-cancer agent, cisplatin. Apoptosis-related protein expression (cleaved caspase-8, cleaved caspase-7, cytochrome c, cleaved caspase-9, cleaved caspase-3, and poly (ADP-ribose) polymerase (PARP) was increased by treating with 5 μM of moscatilin. This suggests that moscatilin-mediated apoptosis is associated with the extrinsic and intrinsic apoptotic signaling pathways. In addition, moscatilin-induced apoptosis was mediated by the c-Jun N-terminal kinase (JNK) signaling pathway. Overall, this study identified additional biological activity of moscatilin derived from natural products and suggested its potential application as a chemotherapeutic agent for the management of head and neck squamous cell carcinoma.

## 1. Introduction

Head and neck squamous cell carcinoma (HNSCC), arising in the oral cavity, larynx, and pharynx, is the sixth most common cancer globally, with approximately 600,000 cases diagnosed annually [1]. Oncogenic stimuli for HNSCC, such as smoking, alcohol consumption, and viral infection, could serve as potential triggers [2]. Despite improvements in clinical therapeutics, such as surgery, chemotherapy, and radiotherapy, the survival rates of patients with HNSCC have not improved over the past 50 years and remain at a 50% five-year survival rate [1,2]. Therefore, it is necessary to develop chemotherapeutic agents that effectively treat HNSCC. In response to this need, several studies have reported natural product-derived compounds with anti-cancer mechanisms in HNSCC [3,4,5].

The stems of several *Dendrobium* species (Orchidaceae) are used as herbal medicines. Dendrobii Herba [6] is used to treat fever, hydrodipsomania, stomach disorders, and amyotrophia in East Asia [7]. The major components in this species are bibenzyl compounds [8,9,10,11,12,13] with diverse biological effects that include anti-inflammatory [8], antioxidant [8], anti-cancer [9,10], retinal neoangiogenesis inhibitory [11], and antimutagenic [12,13] activities.

Recently, in the course of searching for active components with anti-cancer potential from natural products, the ethyl acetate-soluble fraction of Dendrobii Herba showed considerable cytotoxicity against the FaDu human pharyngeal squamous carcinoma cell line. Thus, it was subjected to bioassay-guided fractionation, which led to the isolation of 13 compounds. Among the isolates, moscatilin exhibited significant cytotoxicity against the FaDu cell line [14]. Several studies have reported that moscatilin exerted potent effects on numerous cancer cell lines [15,16,17,18,19]. Moscatilin was shown to induce apoptosis in human colorectal cancer cells through tubulin depolymerization and DNA damage and c-Jun N-terminal kinase (JNK) activation [15], apoptosis of human pancreatic cancer cells via reactive oxygen species and the JNK/stress-activated protein kinases (SAPK) pathway [16], and apoptosis and mitotic catastrophe in human esophageal cancer cells by early promotion of the M phase cell cycle blockade and the regulation of mitotic catastrophe-associated proteins [17]. Moscatilin was also reported to inhibit the migration and metastasis of human breast cancer cells by inhibiting Akt and the Twist signaling pathway [18]. In addition, moscatilin suppressed tumor angiogenesis and growth in human umbilical vein endothelial cells, blocking ERK1/2, Akt, and the eNOS pathway [19].

To the best of our knowledge, the apoptotic mechanism of moscatilin in HNSCC has not been reported, even though its anti-cancer activities involved in cell signaling pathways against various cancer cells have been studied previously. Therefore, in this study, we further investigated the mechanism of action of moscatilin by using A549 human lung cancer cells. Consequently, we further investigated the mechanism of action of moscatilin using FaDu human pharyngeal squamous carcinoma cells.

## 2. Results and Discussion

### 2.1. Moscatilin Induces Death of FaDu Cells Via Increased Cytotoxicity

**Figure 1:**
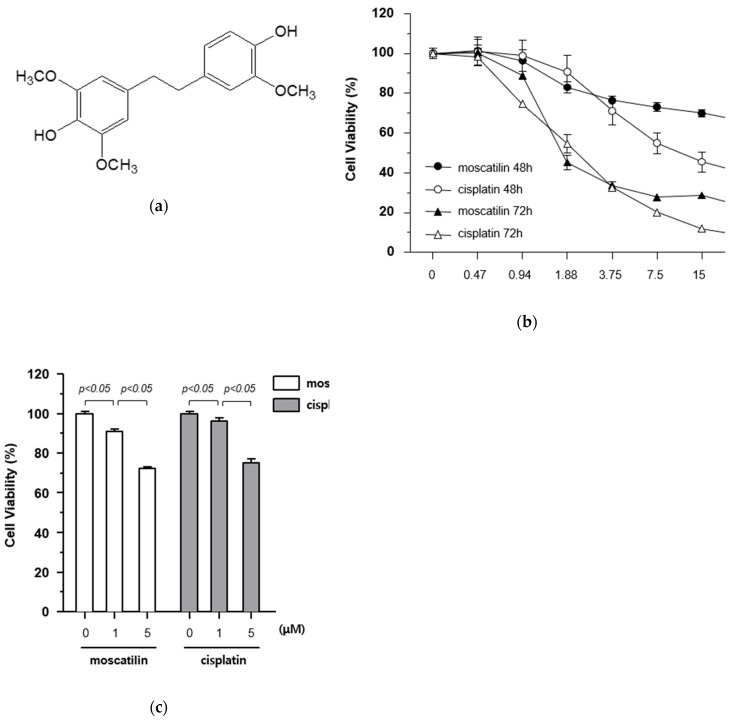
Cytotoxicity of moscatilin on FaDu cells. (**a**) Chemical structure of moscatilin. (**b**) FaDu cells (2 × 10^4^ cells/well) were seeded in 96-well plates and treated with 0.47–30 μM moscatilin or cisplatin for the indicated times. Cell viability was measured using a CCK-8 assay kit. (**c**) Cell viability was measured by the CCK-8 assay kit 48 h after treatment with 1 μM moscatilin, 5 μM moscatilin, or cisplatin for 48 h. The values are expressed as the mean ± SD of five independent experiments.

The cytotoxic effects of moscatilin and cisplatin on FaDu cells were determined using a Cell Counting Kit-8 (CCK-8) assay kit. The cells were treated with varying concentrations of moscatilin and cisplatin (0.47, 0.94, 1.88, 3.75, 7.5, 15, and 30 μM) for 48 and 72 h. The results of the 72 h CCK-8 assays showed that IC_50_ values for moscatilin and cisplatin were 1.418 μM and 1.856 μM, respectively (Figure 1B). As shown in Figure 1B, the treatment of cells with moscatilin doses lower than 3.75 μM for 48 or 72 h showed cytotoxicity similar to that of cisplatin. However, at doses higher than 3.75 μM for 48 or 72 h, the cytotoxicity of moscatilin was lower than those of cisplatin. Subsequently, to determine the exact cytotoxic effect of moscatilin and cisplatin, the viability of FaDu cells was measured at 1 and 5 μM moscatilin and cisplatin. As shown in Figure 1C, the viability of FaDu cells at 1 μM and 5 μM decreased by 9.3% and 27.8% for moscatilin, but only 3.7% and 25.0% for cisplatin, respectively, when compared to the untreated control cells. This suggests that moscatilin had a cytotoxic effect similar to cisplatin in FaDu cells.

Moscatilin (25 μM) has been shown to inhibit cell viability in in various cancer cell lines including osteosarcoma, lung, neuroblastoma, colon, cervical, hepatic, and pancreatic cancers [16]. Compared with these published results, the treatment of a similar concentration of moscatilin in FaDu cells showed similar or higher cytotoxicity in this study.

### 2.2. Moscatilin-Induced Cell Death is Mediated By Apoptosis and Suppresses Cell Proliferation

**Figure 2:**
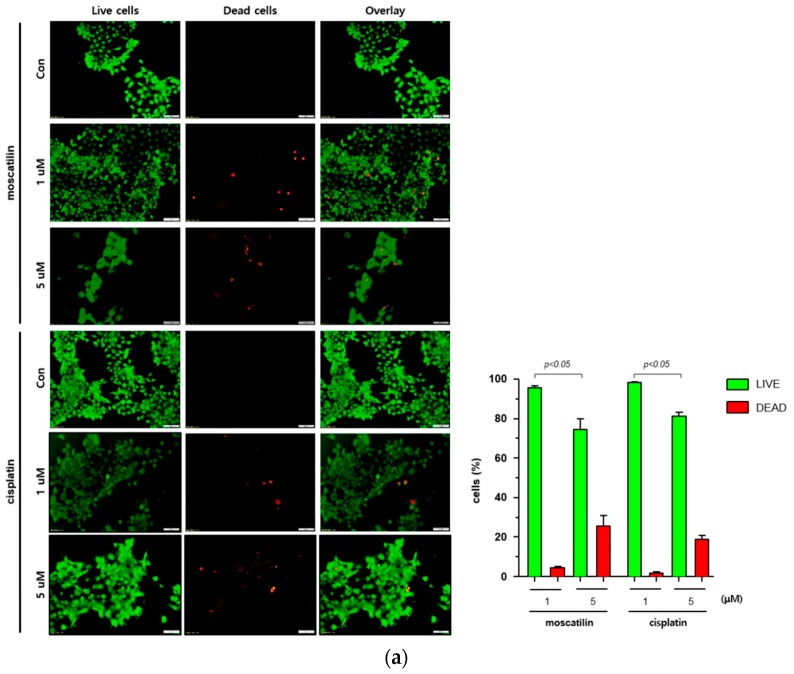

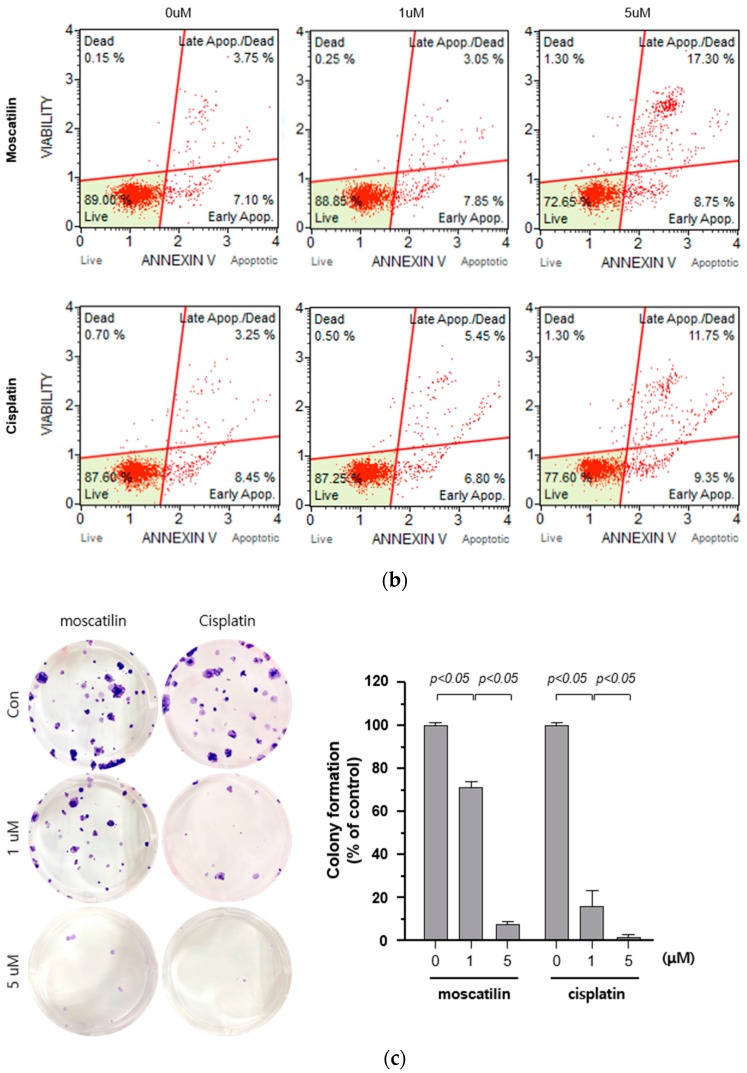
Moscatilin induced cell death is mediated by apoptosis. (**a**) FaDu cells (2 × 10^4^ cells/well) were treated with 1 μM moscatilin, 5 μM moscatilin, or cisplatin for 48 h. The live and dead cells were stained with green calcein-AM and ethidium homodimer-1 (red) and then analyzed using confocal microscopy. Scale bar is 50 μm. The values are expressed as the mean ± SD of five independent experiments. (**b**) FaDu cells (2 × 10^5^ cells) were seeded in six-well plates and treated with 1 μM moscatilin, 5 μM moscatilin, or cisplatin. After a 48 h incubation, the cells were collected, washed twice with ice-cold phosphate-buffered saline (PBS), resuspended in PBS containing 10% FBS, and then stained with annexin V and PI for 20 min. The apoptotic population was analyzed by the Muse Cell Analyzer. (**c**) Equal amounts of FaDu cells were seeded in six-well plates and treated with the indicated concentrations of moscatilin and cisplatin. After a 24-h incubation, the FaDu cells were incubated in culture media without moscatilin or cisplatin for 10 days, stained with crystal violet, and then the colonies were counted. The values are expressed as the mean ± SD of three independent experiments.

Cell death occurs by a variety of causes, including necrosis, apoptosis, or autophagy, and can be analyzed by a variety of methods, including the measurement of dead cells (i.e., cytotoxicity assay) and the quantification of live cells (i.e., viability assay) [20]. Therefore, a single method may not be sufficient to determine the type of cell death. To determine the type of cell death by moscatilin, FaDu cells were treated with 1 or 5 μM moscatilin for 48 h and cell death was measured using a live and dead assay kit. As shown in Figure 2A, treating cells with 5 μM moscatilin for 48 h resulted in a significant increase in cell death compared to 1 μM moscatilin. Next, to investigate whether the cell death by moscatilin was mediated by apoptosis, FaDu cells were treated with 1 and 5 μM moscatilin for 48 h. Flow cytometry (FACS) analysis was also performed using an annexin V/fluorescein isothiocyanate (FITC) assay kit. As shown in Figure 2B, the population of apoptotic cells (early and late apoptotic cells) significantly increased in FaDu cells treated with 5 μM moscatilin compared to 1 μM moscatilin-treated cells. This suggests that moscatilin may have induced cell death through apoptosis in FaDu cells, which is similar to the mechanism of cisplatin. Cisplatin has been used to treat a variety of cancer types, including testicular, ovarian, cervical, head and neck, and non-small-cell lung cancers for multiple years [21]. It is well known that cisplatin used as cancer therapy inhibits proliferation and cell growth and induces cell death [22]. Therefore, the proliferation of FaDu cells after treatment with moscatilin and cisplatin was measured and compared using a colony formation assay. As shown in Figure 2C, the percentage of colony formation at 1 μM and 5 μM moscatilin decreased by about 30% and 90%, respectively, compared to untreated control cells. However, the decrease in cell proliferation by moscatilin was lower than that of cisplatin. This indicates that moscatilin effectively suppressed the proliferation similarly to cisplatin in FaDu cells. Cisplatin is the first-line chemotherapy drug used in HNSCC. However, in some cases, cisplatin leads to a poor prognosis due to drug resistance. It represents the main reason for chemotherapy failure [23]. Since the cytotoxic effect of moscatilin on FaDu cells caused by apoptosis and the suppression of proliferation is similar to that of cisplatin, moscatilin could be an alternative option for cisplatin resistance patients. However, additional research is required to explore the clinical advantages and fully understand the mechanism of moscatilin-induced apoptosis in HNSCC.

### 2.3. Moscatilin Induces the Apoptosis of FaDu Cells Through Extrinsic and Intrinsic Apoptotic Pathways

**Figure 3:**
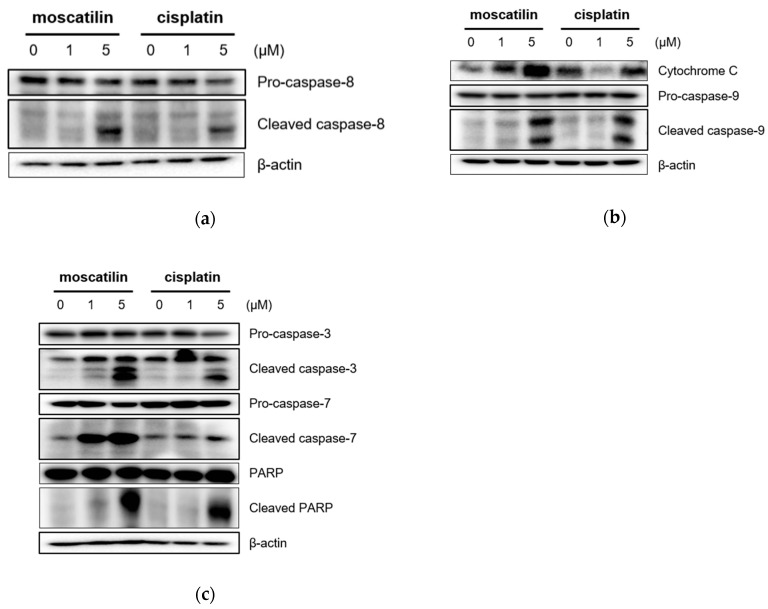
Moscatilin induces the apoptosis of FaDu cells through the extrinsic and intrinsic apoptotic pathways. FaDu cells were treated with 1 μM moscatilin, 5 μM moscatilin, or cisplatin. After a 24-h incubation, the cells were harvested and cell lysates were prepared. The levels of (**a**) pro-caspase-8 and cleaved caspase-8, (**b**) cytochrome C, pro-caspase-9, and cleaved caspase-9, (**c**) pro-caspase-3, cleaved caspase-3, pro-caspase-7, cleaved caspase-7, PARP, and cleaved PARP were compared by Western blotting. β-actin was used as a loading control.

Apoptosis triggered by the caspase-cascade pathway can be initiated either through the extrinsic (death receptor pathway) or the intrinsic pathway (mitochondrial pathway) [24]. The intrinsic pathway is initiated through Bcl-2 protein family members in the outer mitochondrial membrane in response to cell damage and cellular stress stimuli [25]. Following these signals, the cytochrome c released from damaged mitochondria activates caspase-9. The extrinsic pathway initiated through the extracellular stimulation of death receptors, such as the tumor necrosis factor (TNF) or the TNF-related apoptosis-inducing ligand (TRAIL), results in the activation of the initiator caspase-8 [26]. Subsequently, the activation of caspase-3 and caspase-7 by both intrinsic and extrinsic signaling pathways induces the activation of PARP to induce the DNA fragmentation in apoptosis [25,26]. Therefore, to evaluate apoptotic signaling pathways by moscatilin, apoptosis-related protein expression was analyzed using Western blotting analysis. As shown in Figure 3A, cleaved caspase-8, which is an extrinsic marker, increased significantly in FaDu cells treated with moscatilin in a concentration-dependent manner. These data indicate that the extrinsic apoptotic signaling pathway was involved in moscatilin-induced apoptosis in FaDu cells. Furthermore, the expression of cytochrome c and cleaved caspase-9, which are the intrinsic markers, increased similarly by treatment with either moscatilin or cisplatin in concentration-dependent manners, as shown in Figure 3B. These data indicate that the intrinsic apoptotic signaling pathway was involved in both moscatilin and cisplatin-induced apoptosis in FaDu cells. Caspase-3 and PARP are target molecules of both the extrinsic and intrinsic apoptotic signaling pathways [22,24]. Therefore, we analyzed the expression of cleaved caspase-3 and PARP. As shown in Figure 3C, the cleaved caspase-3 and PARP significantly increased by both moscatilin and cisplatin. Taken together, these data suggest that the moscatilin-induced apoptosis in the FaDu cell death was mediated by both the extrinsic and intrinsic apoptotic signaling pathways.

### 2.4. Moscatilin-Induced Apoptosis in FaDu Cells Was Regulated by the JNK Signaling Pathway

**Figure 4:**
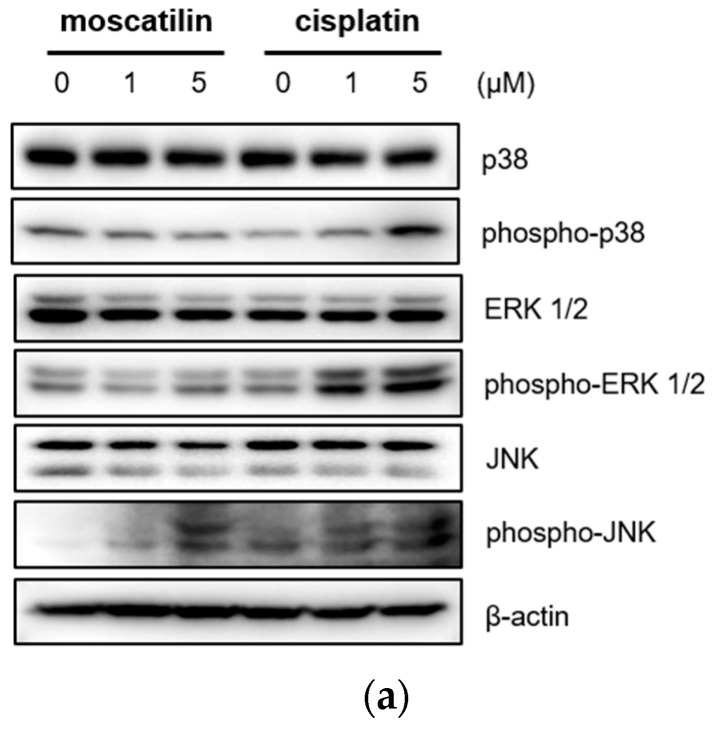

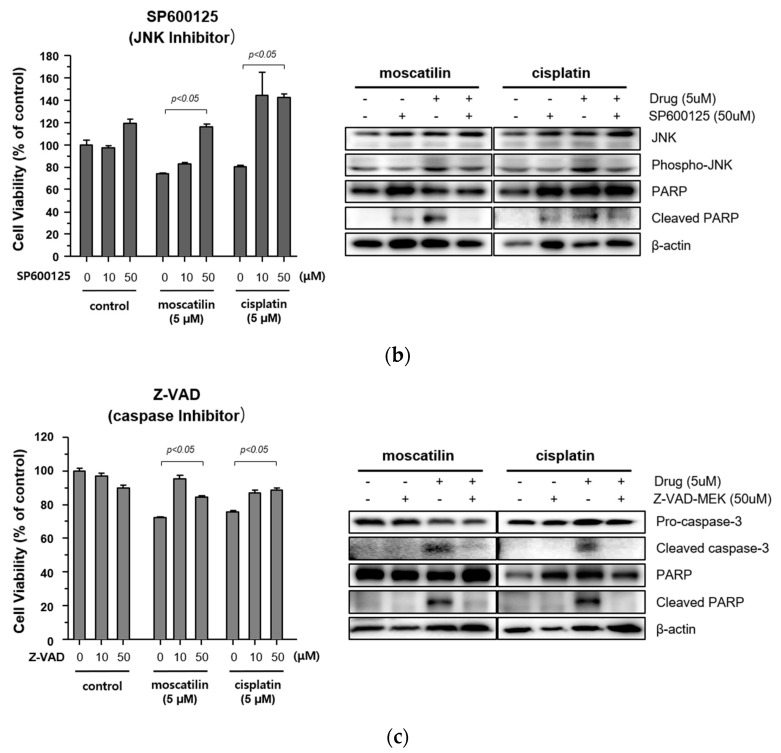
Moscatilin-induced apoptosis in FaDu cells was regulated by the c-Jun N-terminal kinase (JNK) signaling pathway. (**a**) FaDu cells were treated with 1 or 5 μM of moscatilin or cisplatin. After a 24-h incubation, the cells were harvested and cell lysates were prepared. The levels of p35, phosphor-p38, ERK 1/2, phosphor-ERK 1/2, JNK, and phosphor-JNK were compared by Western blotting. β-actin was used as a loading control. FaDu cells were pre-treated with 10 μM and 50 μM of SP600125 (JNK inhibitor) (**b**) or Z-VAD-FMK (a pan-caspase inhibitor) (**c**) for 30 min, prior to treatment with 5 μM moscatilin or cisplatin. The levels of JNK, phosphor-JNK, caspase-3, cleaved caspase-3, PARP, and cleaved PARP, were compared by Western blotting. β-actin was used as a loading control.

Cell proliferation and apoptosis were regulated by the activation of the caspase cascade through a mitogen-activated protein kinase (MAPK) signaling pathway [27]. The MAPK family is comprised of p38 mitogen-activated protein kinase (p38 MAPK), extracellular signal-regulated kinase (ERK), and Jun kinase (JNK/SAPK) [28]. Previous studies also showed that moscatilin induced apoptotic cell death by activating the c-Jun N-terminal kinase (JNK) in colorectal and pancreatic cancer cells [15,16]. Therefore, to identify the cellular signaling mechanism responsible for caspase activation by moscatilin and cisplatin, the protein expression levels of p38 MAPK, ERK1/2, and JNK after treatment with 1 μM moscatilin, 5 μM moscatilin, or cisplatin for 48 h were determined by Western blotting. As shown in Figure 4A, the expression levels of p38, ERK ½, and JNK following treatment of FaDu cells with cisplatin were similar and the expression levels of phosphorylated p38, ERK ½, and JNK increased significantly in dose-dependent manners. However, the expression levels of phosphorylated JNK only increased significantly by treatment with moscatilin. Next, to confirm whether the moscatilin-induced apoptosis was mediated by the JNK signaling pathway, FaDu cells were treated with both moscatilin and a JNK inhibitor, SP600125. The JNK inhibitor blocked the moscatilin-induced activation of JNK and PARP as well as the attenuated moscatilin-induced cytotoxic effects, as shown in Figure 4B. The end-stage of apoptotic signaling through the phosphorylation of MAPKs, such as JNK, is mediated by activating caspase-3 and PARP [29]. To investigate whether the moscatilin-induced apoptosis in FaDu cells was dependent on the activation of caspase, FaDu cells were co-treated with 5 μM moscatilin and 50 μM Z-VAD-fmk, which is a pan-caspase inhibitor. A CCK-8 assay was then performed to measure cell viability, and Western blotting was used to observe changes in the expression of cleaved caspase-3 and PARP. As shown in Figure 4C, 5 μM of moscatilin decreased the viability of FaDu cells by about 45% when compared to untreated control cells. However, the viability of FaDu cells was partially attenuated in the presence of Z-VAD-fmk and moscatilin. Furthermore, the moscatilin-induced upregulation of cleaved caspase-3 in FaDu cells decreased by Z-VAD-fmk. Subsequently, Z-VAD-fmk significantly suppressed moscatilin-induced cleavage of the pro-form of PARP in FaDu cells. Therefore, these data suggest that the moscatilin-induced apoptosis in FaDu cells was initiated by activating the caspase cascade through the phosphorylation of JNK signaling pathways. In addition, since moscatilin induced apoptosis via different apoptotic signaling pathways when compared to cisplatin, the combination of moscatilin with the chemotherapy drug cisplatin could be a new strategy for chemotherapy to increase the antitumor response. However, to explore the potential for their synergistic effects on HNSCC, further anti-cancer mechanism studies in vitro and in vivo are required.

## 3. Materials and Methods

### 3.1. Reagents

The following antibodies were used in this study: anti-caspase-3, anti-caspase-7, anti-Caspase-8, anti-caspase-9, anti-ERK1/2, anti-phospho-ERK1/2, anti-JNK, anti-phospho-JNK, anti-p38, anti-phospho-p38, and anti-PARP from Cell Signaling Technology (Danvers, MA, USA), and anti-β-actin from Santa Cruz Biotechnology (Santa Cruz, CA, USA). SP600125, SB203580, PD98059, and Z-VAD-FMK were purchased from Tocris Bioscience (Tocris Bioscience, Bristol, UK).

### 3.2. Cell Culture and Treatments

The FaDu (human pharyngeal squamous carcinoma cell) cell line was purchased from the American Type Culture Collection (ATCC, Manassas, VA, USA). The FaDu cells were cultured in Minimum Essential Medium (Corning Life Sciences, CorningNY, USA), supplemented with 10% fetal bovine serum (FBS; Hyclone, Logan, UT, USA), and incubated at 37 °C in a 5% CO_2_ incubator.

### 3.3. Cell Viability Assay

To determine the viability of the FaDu cells, a CCK-8 assay kit (Dojindo, Kumamoto, Japan) was used according to the manufacturer’s protocol. The FaDu cells were seeded into 96-well plates at a density of 2 × 10^4^ cells/well and incubated at 37 °C for 24 h. After incubation, the cultured FaDu cells were treated with varying concentrations of moscatilin (0.47–30 μM) for 48 h. Thereafter, 10 μL of the CCK-8 reagent was added into the cultured FaDu cells and incubated for a further 4 h. The absorbance was measured at 450 nm using a SPARK^®^ multimode microplate reader (Tecan, Männedorf, Switzerland). The 50% inhibitory concentration (IC_50_) was calculated from a dose-response analysis performed using GraphPad Prism software (GraphPad Software, La Jolla, CA, USA).

### 3.4. Live and Dead Cell Assay

To visualize the live and dead FaDu cells, the LIVE/DEAD^®^ Viability/Cytotoxicity Kit (Thermo Scientific, Rockford, IL, USA) was used according to the manufacturer’s protocol. The FaDu cells were seeded into 35-mm confocal dishes at a density of 0.2 × 10^5^ cells/mL and incubated at 37 °C for 24 h. After incubation, the cultured FaDu cells were treated with the indicated concentrations of moscatilin or cisplatin (0, 1, and 5 μM) for 48 h. Thereafter, 5 μL of calcein-AM and 20 μL of ethidium homodimer-1 reagent were added into cultured FaDu cells, and the cells were incubated for a further 0.5 h. The stained cells were analyzed by confocal microscopy.

### 3.5. Apoptosis Assay

To determine the extent of the apoptosis and necrosis of the FaDu cells, a Muse^TM^ Annexin & Dead Cell Kit (Merck Millipore, Billerica, MA, USA) was used according to the manufacturer’s protocol. The FaDu cells were seeded into six-well plates at a density of 2 × 10^5^ cells/well and incubated at 37 °C for 24 h. After incubation, the cultured FaDu cells were treated with the indicated concentrations of moscatilin or cisplatin (0, 1, and 5 μM) for 48 h, harvested using 0.05% trypsin/EDTA, and washed two times with cold PBS by centrifugation at 3000 rpm for 3 min. Thereafter, the harvested cells were resuspended with 2 mL of PBS containing 10% FBS and 100 μL of the suspension was transferred to a new tube. Then 100 μL of the Muse^TM^ Annexin & Dead Cell Kit reagent was added. After incubation in the dark for 20 min, the samples were analyzed using a Muse Cell Analyzer (Merck Millipore).

### 3.6. Colony Formation Assay

A colony formation assay was performed according to a previously described protocol [30]. FaDu cells were cultured at a density of 200 cells/well in six-well culture plates and incubated at 37 °C for 24 h. After incubation, the cultured FaDu cells were treated with 1 and 5 μM dendrophenol or cisplatin for 24 h and then incubated in a culture medium without moscatilin or cisplatin for 14 days. Thereafter, the medium was removed, and the cells were washed with PBS. Sequentially, the colonies were stained with 2% crystal violet for 30 min, washed with PBS, and dried at room temperature. Photographs of the stained colonies were taken by a digital camera and analyzed.

### 3.7. Western Blotting

Cell lysates were prepared using previously described methods [31]. Equal amounts of cell lysate were separated using SDS-PAGE, electroblotted onto a nitrocellulose filter (Millipore), and analyzed using the specified antibodies with an ECL detection system (BD Biosciences, San Diego, CA, USA).

### 3.8. Statistical Analysis

All experiments were performed at least three times to obtain means and standard deviations. Statistical significance was determined with one-way analysis of variance using GraphPad Prism software (GraphPad Software, La Jolla, CA, USA). *p*-values less than 0.05 were considered significant.

## 4. Conclusions

Our study demonstrated that moscatilin induces apoptosis of human pharyngeal squamous carcinoma cells by activating caspases through the JNK signaling pathway. The present study provided an additional mechanism of action on the pro-apoptotic effect of moscatilin and supported its application as a cancer chemotherapeutic agent for HNSCC.
